# Opportunities and obstacles to screening for perinatal depression among women in Zimbabwe: A narrative review of literature

**DOI:** 10.4102/sajpsychiatry.v24i0.1127

**Published:** 2018-08-28

**Authors:** James January, Moses J. Chimbari

**Affiliations:** 1 Department of Psychiatry, University of KwaZulu-Natal, South Africa; 2 School of Nursing and Public Health, University of KwaZulu-Natal, South Africa

## Abstract

**Background:**

The perinatal period provides an opportune time for health care providers to screen for and proffer interventions for women suffering from depression. However, routine screening for depression is not done in primary care settings in Zimbabwe.

**Aim:**

This narrative review discusses opportunities and obstacles surrounding screening for perinatal depression in primary care settings in Zimbabwe, with a view to stress the importance of routine screening to policy-makers.

**Methods:**

Both electronic and manual searches were done on PubMed, PubMed Central, African Journals Online, Google Scholar and the University of Zimbabwe Institutional Repository (UZIR) using the following key terms: ‘women and antenatal depression’, ‘prenatal depression’, ‘postnatal depression’, ‘postpartum depression’, ‘depressive disorder’, or ‘common mental disorder’ and ‘screening and Zimbabwe’.

**Results:**

Although opportunities for depression screening are possible because of the high antenatal and postnatal service coverage, the potential for universal screening is fraught with human and financial resource constraints, lack of training in mental health care among primary health care providers and lack of locally validated screening tools for depression.

**Conclusion:**

There is a need to channel resources into the training of midwives and other primary health care providers on mental health issues affecting women perinatally.

## Introduction

Depression exerts a substantial burden on women during the perinatal period.^1^ In a recent systematic review, it was shown that antenatal depression may have short- and long-term negative effects on the foetus, newborns and adolescents.^2^ Evidence suggests that women with perinatal depression tend to have children who are undernourished,^3^ have adjustment difficulties,^4^ are stunted,^5^ are poorly fed, are less likely to be vaccinated and experience compromised safety practices.^6^ Alder and colleagues reported that pregnant women with elevated levels of depressive symptoms were more likely to experience obstetric complications and preterm labour.^7^ All these potential adverse outcomes warrant more concerted efforts to tackle this often neglected condition, especially in resource-poor settings.

The Global Burden of Diseases Report of 2010 showed that major depressive disorders (MDD) were the second leading cause of years lived with disability (YLD) with a prevalence of 8.2%. The largest proportion of these depressive disorders occur between the ages of 15 and 64 years with women being the most affected.^8^ Women in low- and middle-income countries (LMICs) are disproportionately affected by depression with prevalence of postnatal depression (PND) as measured by screening studies using the Edinburgh Postnatal Depression Scales (EPDS), reaching up to 34% in Zimbabwe.^9,10,11^ In Zimbabwe, common mental disorders (CMDs) like depression and anxiety are prevalent among women.^12^ Recently, it was reported that 21.4%, 21.6% and 4% of respondents to a survey conducted in Harare had PND as measured by the Center for Epidemiological Studies-Depression Scale, postpartum suicidal ideation and suicidal attempts, respectively, during the perinatal period.^13^ Challenges, however, exist in estimating the true burden of perinatal depression. This is in part because of the fact that there is a paucity of studies using diagnostic criteria such as the Structured Clinical Interview for Diagnostic and Statistical Manual of Mental Disorders (SCID). Additionally, most studies in Zimbabwe on perinatal depression are carried out in urban settings and scant data exist for rural populations.^11,14^

Studies that have been conducted in Zimbabwe have indicated that factors which increase the risk of PND include HIV, gender, socio-economic status and quality of life.^14^ In a study carried out in 2016 among 264 adults, people living with HIV were found to have a higher prevalence of possible CMDs (67.94%) and depression (68.5%) than those without HIV (CMDs – 51.4%; depression – 47.2%).^15^ Common mental disorders and depression were also associated with being female (72.6%) and experiencing negative life events like divorce, intimate partner violence and unemployment.^15^

Because of lack of free-standing mental health facilities in most parts of Zimbabwe, psychological services are often integrated into primary care. Antenatal and postnatal visits provide some of the most ideal opportunities for health care providers to recognise, screen for and possibly respond to women who may be having depressive symptoms, and thereby potentially enhance pregnancy outcomes. The recently completed Demographic and Health Survey for Zimbabwe indicated that nearly all (93%) women who had given birth in the 5-year period preceding the survey had received antenatal care (ANC) from a trained health care professional at least once.^16^ This presents an ideal opportunity to potentially screen women for depression during pregnancy. Additionally, a substantial percentage of women (76%) had had 4 or more ANC visits, which reflected a good increase from the 65% reported in the 2010–2011 survey. There were also no major differences in these percentages between urban and rural women.

## Benefits of screening for perinatal depression

The United States of America Preventive Services Task Force recently concluded that depression screening programmes for pregnant and postnatal women may have a positive effect of reducing prevalence of depressive symptoms.^1^ If screening is followed by effective treatment, the burden of depression among women in the perinatal period can be significantly reduced. Thus, screening provides a window of opportunity to detect women requiring further diagnosis and treatment. Furthermore, screening may encourage women to open up and talk about their problems, resulting in less stigma surrounding depression.

In light of the foregoing arguments, this review of literature aimed at determining the existing and potential opportunities and obstacles to screening for perinatal depression among women in Zimbabwe.

## Methods

**FIGURE 1:**
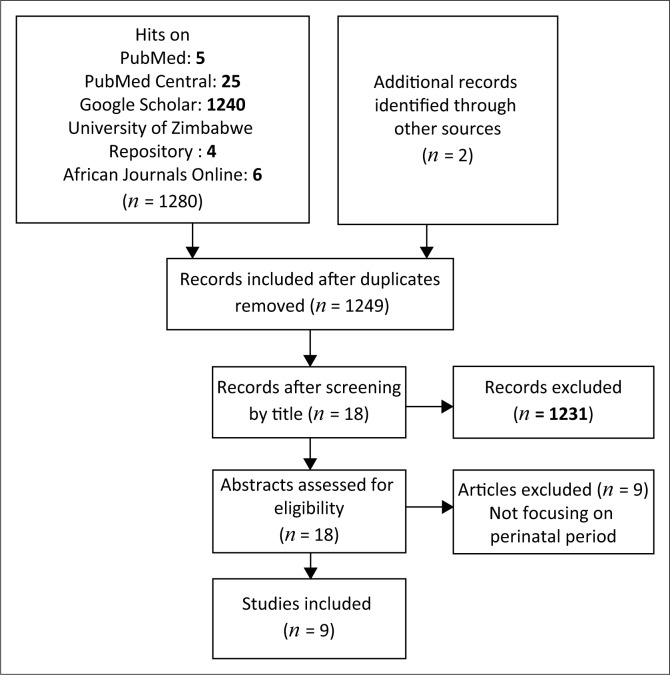
Flow diagram on how searches were conducted.

We synthesised literature retrieved from studies conducted in Zimbabwe focusing on screening for perinatal depression among women. No time restrictions were imposed on the search. Two independent librarians searched the following electronic databases: PubMed, PubMed Central, African Journals Online, Google Scholar and the University of Zimbabwe Institutional Repository (UZIR) (Figure 1). We employed combinations of the medical subject heading terms: *women* and *antenatal depression, prenatal depression, postnatal depression, postpartum depression, depressive disorder,* or *common mental disorder* and *screening* and *Zimbabwe.* Inspection of bibliographic lists of retrieved articles was conducted to identify any additional relevant articles. Only articles written in the English language and which reported results from Zimbabwe were included. No restrictions were imposed on the types of studies included in the review. Results from the review were grouped into two main thematic areas: (1) opportunities for screening and (2) challenges faced in screening.

## Ethical consideration

Ethical approval was obtained from the University of KwaZulu-Natal’s Biomedical Research Ethics Committee (BREC # BE 598/16).

## Results and discussion

### Search results

Electronic searches using PubMed yielded five journal articles. A total of 25 articles were identified using PubMed Central, 6 articles were found using African Journals Online (AJOL), 4 (1 journal article and 3 dissertations) were found on the UZIR and 1240 articles were identified through Google Scholar. After screening the articles by title where we excluded all non-Zimbabwean studies, 5 remained from PubMed, 7 from PubMed Central, 3 from Google Scholar, one from UZIR and none from AJOL. A description of the resultant 9 reviewed studies published between 1998 and 2017 is provided in Table 1.

**TABLE 1:** Summary of reviewed studies.

Author and year	Target population	Sample size	Study design	Setting
Nhiwatiwa et al. 1998	Prenatal and postnatal women	500	Prospective cohort study	Peri-urban-Epworth, Harare
Stranix-Chibanda et al. 2005	Common mental disorders	437	Cross-sectional study	Peri-urban Chitungwiza
Chibanda et al. 2010	Postnatal women	210	Validation study	Urban-Chitungwiza
Chibanda et al. 2010	Postnatal women	210	Cross-sectional study	Urban-Chitungwiza
Chibanda et al. 2014	Postnatal women	210	Randomised control trial	Primary care urban clinics
January et al. 2015	Postnatal women	295	Cross-sectional study	Urban-Harare
Shamu et al. 2016	Postnatal women	842	Cross-sectional study	Urban-Harare
January et al. 2017	Postnatal women	115	Cross-sectional study	Peri-urban and rural
January et al. 2017	Postnatal women	5	Scoping review	Zimbabwe

### Opportunities for screening women for depression

As previously discussed, the primary care setting offers a good opportunity for women to be screened for depression in Zimbabwe. As there is good ANC coverage, the health care system could take advantage of the high numbers of women who attend ANC services and offer screening. Health care workers could sensitise women on perinatal depression during health promotion sessions in primary care settings when women present for ANC or for other primary care services. Targeting women for potential screening when they attend for ANC may however not be feasible for sections of society that have lower odds of utilisation of health services such as Apostolics.^17^ Previous studies from Zimbabwe reported that Apostolics consider the church as an alternative health care system.^18,19^ This indicates that community-based screening programmes involving both village health workers and religious healers may improve coverage. The cost of using lay health workers to screen for perinatal depression in communities is relatively low as compared to using nurses.^20,21^ Periodic supportive supervision will however be required to ensure that the assessments are conducted in a standardised manner.

The postnatal period can also be used to screen for those women who may have missed screening or screened negative at the ANC stage. However, challenges would exist in effectively reaching most women following childbirth as most provinces within Zimbabwe reported postnatal care (PNC) coverage of less than 80% with 4 out of the 10 provinces reporting less than 50% (see Table 2). In light of these PNC coverage levels, using community-based screening in addition to health-facility-based screening would potentially yield better results.

**TABLE 2:** Maternal health services across provinces in Zimbabwe.

Author to provide heading	Mashonaland East	Mashonaland West	Mashonaland Central	Matebeleland North	Matatebeleland South	Midlands	Manicaland	Masvingo	Harare	Bulawayo
At least four ANC visits	74.4	80.1	77.2	77.7	80.8	77.2	68.7	78	73.3	78.7
Health facility delivery	66.9	67.5	71.2	62.6	81	81.9	75.7	69.9	78.9	84.1
PNC attendance	41.4	47.3	54.5	53.5	72.5	79.4	57.3	36.1	49.9	60.2

*Source*: ZDHS^16^

ANC, antenatal care; PNC, postnatal care.

Another important yet often neglected entry point involves using traditional or religious health care practitioners. Pregnant women in Zimbabwe have been reported to also seek help from non-medical practitioners.^22^ This provides an opportunity for integrating activities of traditional medical practitioners with their biomedical counterparts.^18,23^ Such integration would contribute towards increasing awareness of mental health problems that women may suffer during the perinatal period so that appropriate interventions can be instituted. Furthermore, to optimise benefits to women who engage in medical pluralism, especially in resource-limited settings such as Zimbabwe, there is need to address structural and cultural realities of screening for perinatal depression. Thus, more studies exploring these cultural antecedents are warranted. There is also need to establish proper referral patterns for women identified to be at higher risk for depression. Thus, future studies could potentially explore whether traditional medical practitioners would be willing to refer women they identify as having depression to biomedical practitioners and vice versa.

Based on lessons learnt from HIV programmes where provider-initiated counselling and testing has yielded positive results,^24^ a similar approach may also be instituted regarding mental health screening. Thus, use of multi-component health programmes has the potential to yield maximum health benefits for primary care attenders. However, multi-component interventions would require appropriate training, more financial support and provision of locally developed and culturally relevant screening tools such as the Shona Symptom Questionnaire (SSQ) for mental health problems. Furthermore, the success of such approaches hinges heavily on their incorporation into existing policy and legislative frameworks. To minimise the amount of time taken when screening for depression, health care workers in Zimbabwe can adopt shorter versions of screening instruments such as the two-item Patient Health Questionnaire (PHQ-2) which is administered as an initial brief screening for depression. The PHQ-2 has been found to have a high sensitivity of 91% when it was validated in a community with high HIV prevalence in Zimbabwe.^25^ Additionally, the existence of the SSQ which is an indigenous measure of CMD also presents an ideal opportunity to screen women during the perinatal period using emic criteria. Previous studies have utilised this screening measure to estimate the prevalence of CMDs in prenatal and postnatal women.^26^

### Challenges in screening for postnatal depression among women

The health care sector in Zimbabwe has, in recent years, suffered as a result of the economic downturn that is being experienced in the country. There has been an increase in outmigration of health workers, particularly nurses,^27^ and that has resulted in depleted clinical staff levels.^28^

To compound the problem of depleted clinical staff, the few primary care nurses currently do not have adequate training in screening for mental health problems.^28^ It is therefore imperative to train primary health care workers to identify and manage CMDs among women.^25^ Because of staff shortages and heavy workloads, nurses may not have sufficient time to check for mental health problems at primary care facilities. Closely related to this, most patients who present at primary care facilities report somatic complaints and may not highlight the psychological distress they may be suffering.^14^ Most primary care facilities may also lack privacy and as such clients may be less inclined to highlight the mental health distress they may be suffering from. In addition, stigma around mental illness still exists in Zimbabwe,^29^ and this may result in patients not consulting or disclosing that they have psychological distress. The increased burden of caring for a mentally ill relative may also hinder health seeking as family may experience both psychological and economic strain which is likely to result in them seeking spiritual or traditional support rather than biomedical care.^29^

Referral systems for those suffering from depression in Zimbabwe are in most cases poor and unreliable.^28^ This means that only the most severe cases of mental illnesses reach qualified mental health practitioners. There are currently no existing policies in relation to mental health screening among women during the perinatal period, which may make it difficult to institute appropriate interventions. Furthermore, there is need for adequate provision of interventions for those women who need support.

Validation of screening tools for perinatal depression in Zimbabwe has been done mainly for Shona-speaking women who are resident in urban settings.^9,25^ Data are currently unavailable for rural women and those who are not Shona speakers. Thus, there is need for more validation studies on screening tools such as the locally developed SSQ, the EPDS and the PHQ among rural women and other ethnic groupings within the country. Additionally, there is need to develop more locally relevant depression screening tools that will take into account other local languages spoken in Zimbabwe.

Women’s own beliefs and previous experiences regarding mental illness may contribute to challenges surrounding screening for depression.^14^ This is especially so where depressive symptoms may be viewed as normal in pregnancy and following childbirth. Consequently, depression may be overlooked during the perinatal period because its symptoms are often similar to bodily experiences that are associated with pregnancy.^30^ In the absence of reliable data on the acceptability of women to undergo universal perinatal screening for CMDs in Zimbabwe, there is need to carry out large-scale studies so as to inform policy regarding maternal mental health.

### Limitations of the review

Because of the limited studies we found on screening for perinatal depression within Zimbabwe, we did not perform quality assessment of the studies retrieved as we aimed at including as many papers as possible in this narrative review. Furthermore, our results may be biased because of limited published studies on screening for depression within Zimbabwe as there could be some grey literature which was not included in this review. The review also did not include expert opinion from health authorities and focused only on Zimbabwe which limits generalisability of results to other LMICs. Although the search terms included ‘common mental disorders’, the review only focused on perinatal depression and did not include studies on anxiety or other psychological morbidity. However, important lessons learnt from such studies can also be relevant to women with perinatal depression.

## Conclusion

In view of the high rates of PND of between 20% and 34% reported in Zimbabwe,^9,10,11,13^ as compared to high-income settings where it has been estimated at around 10%,^31^ there is need for universal screening of perinatal depression. This review presented a cursory analysis of the opportunities and obstacles surrounding screening for perinatal depression among women in Zimbabwe. It identifies opportunities as well as the multiple-layered complexities surrounding screening for depression during pregnancy and the postnatal period in a resource-limited setting. Our review indicates the need for large epidemiological and qualitative studies aimed at establishing the extent of perinatal depression burden and health provider, and women’s views of the barriers and opportunities for perinatal depression screening. The success of integrating perinatal mental health interventions into primary health care requires support through health policy as well as adequate human and financial resources. Future studies could also consider expanding the review to include other psychological conditions and countries in LMICs.
